# Advances in Medical Education

**Published:** 1880-09

**Authors:** 


*Advances in Medical Education.*—The Medical College of Virginia, at Richmond, and the Medical Department of the University of Virginia at Charlottesville, are instituting a new departure in the course of study, following the example of other schools that recognize the fact that a few short months is insufficient to acquire a medical education. The session of the first named school is extended to *nine months*, and attendance on two full courses of graded lectures required; and in the University of Virginia graded courses will be instituted and also two full courses of lectures of nine months, with an intermediate examination on the elementary branches at the end of the first course.

The medical schools of the country are realizing slowly that not only is a more extended course of study necessary in order to qualify students for graduation, but that in order to develope the student to the best advantage a *graded course of study* is useful. The greater Universities all seem falling into line in this direction, and doubtless it will be forced upon the medical schools at large by that kind of pressure which is indeed the only effective kind—outside pressure; the schools being in the main what the public desire them to be.

It may be too early to hope for something like this in Dental Colleges, as the whole system of teaching in these is new, and in some respects crude, but it may not be amiss to reflect that this is the only way to perfect culture. He would be an unobservant teacher who has not realized over and often how crude is the material which comes to him for manufacture into dentists, and how his efforts have been crippled and thwarted by the lamentable lack of preparation for the more abstruse facts and doctrine he teaches, by the failure of preparation for their reception. Attention untrained, elementary culture neglected, knowledge of basal principles sadly wanting, the teacher feels that his hands are tied, and that his best efforts cannot develope this very raw material into reputable fabrics. A dependence upon the previous training of a preceptor is more than useless, often mischievous, for too often a wrong direction, if any at all, is given to the mind, and errors are to be unlearned; more difficult often than a new teaching. The usefulness to the teacher's mind, of a sort of preparatory department is only too plain, and some diligent minds are already at work in planning how this may be done. It is too early perhaps to say that a definite classification of studies should be made, or the seniors and juniors should be separately lectured to; but it might be well to make it a subject of study on the part of those who have charge of the schools as well as those outside, who aid in shaping public opinion. It has been the fate of the writer within the past few months to meet and question a young man who for several years has been in the office of an intelligent and successful dentist as a student, a youth whose knowledge of practical dentistry was fair, and yet who did not know what was above or below the diaphragm, or in fact that there was such a division of his interior into cavities. It is certainly dull and uphill work to enter on a course of lectures on histology for example, with the knowledge that such material is present before one. Better preparation is essential, and should be sought for.

